# IL-1**β** Suppresses the Formation of Osteoclasts by Increasing OPG Production via an Autocrine Mechanism Involving Celecoxib-Related Prostaglandins in Chondrocytes

**DOI:** 10.1155/2009/308596

**Published:** 2010-02-24

**Authors:** Yusuke Watanabe, Aki Namba, Yukiko Aida, Kazuhiro Honda, Hideki Tanaka, Naoto Suzuki, Hideo Matsumura, Masao Maeno

**Affiliations:** ^1^Nihon University Graduate School of Dentistry, Tokyo 101-8310, Japan; ^2^Department of Fixed Prosthodontics, Nihon University School of Dentistry, 1-8-13, Kanda Surugadai, Chiyoda-ku, Tokyo 101-8310, Japan; ^3^Division of Advanced Dental Treatment, Dental Research Center, Nihon University School of Dentistry, Tokyo 101-8310, Japan; ^4^Department of Oral Health Sciences, Nihon University School of Dentistry, Tokyo 101-8310, Japan; ^5^Department of Biochemistry, Nihon University School of Dentistry, Tokyo 101-8310, Japan; ^6^Division of Functional Morphology, Dental Research Center, Nihon University School of Dentistry, Tokyo 101-8310, Japan

## Abstract

Elevated interleukin (IL)-1 concentrations in synovial fluid have been implicated in joint bone and cartilage destruction. Previously, we showed that IL-1*β* stimulated the expression of prostaglandin (PG) receptor EP4 via increased PGE_2_ production. However, the effect of IL-1*β* on osteoclast formation via chondrocytes is unclear. Therefore, we examined the effect of IL-1*β* and/or celecoxib on the expression of macrophage colony-stimulating factor (M-CSF), receptor activator of NF-*κ*B ligand (RANKL), and osteoprotegerin (OPG) in human chondrocytes, and the indirect effect of IL-1*β* on osteoclast-like cell formation using RAW264.7 cells. OPG and RANKL expression increased with IL-1*β*; whereas M-CSF expression decreased. Celecoxib blocked the stimulatory effect of IL-1*β*. Conditioned medium from IL-1*β*-treated chondrocytes decreased TRAP staining in RAW264.7 cells. These results suggest that IL-1*β* suppresses the formation of osteoclast-like cells via increased OPG production and decreased M-CSF production in chondrocytes, and OPG production may increase through an autocrine mechanism involving celecoxib-related PGs.

## 1. Introduction

Bone remodeling in joints involves the synthesis of bone matrix by osteoblasts and its resorption by mature osteoclasts. Many cytokines and factors, such as interleukin (IL)-1, macrophage colony-stimulating factor (M-CSF), and receptor activator of NF-*κ*B (RANK) ligand (RANKL), are known to induce the differentiation of monocytes/macrophages into osteoclasts; whereas osteoprotegerin (OPG), a soluble decoy receptor for RANKL that competes with RANK for RANKL binding, is known to suppress differentiation [1, 2]. RANKL is highly expressed on osteoblast/stromal cells, primitive mesenchymal cells surrounding the cartilaginous anlagen, and hypertrophying chondrocytes [3]. M-CSF and OPG are produced and released by activated osteoblasts, chondrocytes, and fibroblasts [2, 4–6]. 

IL-1 plays key roles in amplifying and perpetuating inflammation [7] and in the degradation of bone and cartilage in joints [8]. Two IL-1 subtypes have been identified: IL-1*α* and IL-1*β*. An elevated concentration of IL-1 in the synovial fluid has been implicated in joint bone and/or cartilage destruction [9]. In addition, IL-1*β* is closely associated with the expression of M-CSF, RANKL, and OPG in osteoblasts and chondrocytes. IL-1 increases RANKL expression in osteoblasts and chondrocytes [1, 10]. In osteoblasts, IL-1*α* stimulation prompted M-CSF production [2]; whereas the effect of IL-1 on M-CSF expression in chondrocytes is unclear. Conflicting results have been reported regarding the effect of IL-1 on OPG expression, mostly likely because different cells were used in each study [1, 2, 5].

We postulate that an imbalance in bone and cartilage metabolism is responsible for arthrosis of the temporomandibular joint (TMJ). Arthrosis of the TMJ is accompanied by symptoms such as pain and joint sound during jaw movement [11], and results from an imbalance between predominantly chondrocyte-controlled anabolic and catabolic processes. It is characterized by the progressive degradation of components of the articular cartilage extracellular matrix and is associated with inflammatory factors [12–14]. Several studies [15–18] have found elevated levels of the cytokine IL-1*β* in synovial fluid samples obtained from TMJ patients with osteoarthritis, and elevated concentrations of IL-1*β* in synovial fluid have been implicated in joint cartilage destruction. For these reasons, we attempted to clarify the effect of IL-1*β* on chondrocyte function.

Previously, we showed that IL-1*α* suppressed the expression of cartilage matrix proteins through suppression of the autocrine action of bone morphogenetic protein (BMP)-2 using a human chondrosarcoma cell line, OUMS-27 [19]. We also showed that IL-1*β* stimulated cartilage matrix turnover by increasing mainly matrix metalloproteinase-13 production in human chondrocytes [20]. Furthermore, IL-1*β* promoted an imbalance in cartilage matrix turnover through increased inflammatory cytokine production in human chondrocytes [21]. In contrast, IL-6 and soluble IL-6 receptor appeared to suppress the differentiation of chondrocytes and induce the repair of arthrodial cartilage through the increased expression of cartilage matrix proteins, bone morphogenetic protein (BMP)-7, and BMP receptors in human chondrocytes [22]. In addition, we recently reported that IL-1*β* increased the production of prostaglandin E_2_ (PGE_2_), cyclooxygenase-2 (COX-2), and prostaglandin receptor EP4 in human chondrocytes, suggesting that IL-1*β* may promote the expression of EP4 by increasing PGE_2_ production in chondrocytes [23].

However, the effect of IL-1*β* on the formation of osteoclasts via chondrocytes remains unclear. For this reason, we examined the effects of IL-1*β* and celecoxib, a specific inhibitor of COX-2 [24, 25], on the expression of M-CSF, RANKL, and OPG in human chondrocytes, and the indirect effect of IL-1*β* on the formation of osteoclast-like cells using RAW264.7 cells as osteoclast precursors.

## 2. Materials and Methods Real-Time PCR

Chondrocytes were incubated in DMEM containing 10% (v/v) FBS with 0, 10, or 100 U/mL IL-1*β*, with or without 1 *μ*M celecoxib for up to 28 days. Total RNA was isolated from the cells on days 1, 7, 14, 21, and 28 using an RNeasy Mini Kit (QIAGEN, Valencia, CA, USA). The mRNA levels were normalized using a human *β*-actin competitive PCR kit (TaKaRa Bio, Shiga, Japan). The mRNA was converted into cDNA using a RNA PCR kit (GeneAmp; Perkin-Elmer, Branchburg, NJ, USA).

The cDNA mixtures were diluted 1 : 5 in sterile distilled water, and 2-*μ*L aliquots were subjected to real-time PCR using SYBR Green I dye (BioWhittaker Molecular Applications, Rockland, ME, USA) in 25-*μ*l reactions containing 1× R-PCR buffer, 1.5 mM dNTP mixture, 1× SYBR Green I, 15 mM MgCl_2_, 0.25 U Ex Taq (R-PCR version; TaKaRa Bio), and 0.2 *μ*M primers (sense and antisense), as shown in Table 1. PCR primers were designed using Primer3 software (Whitehead Institute for Biomedical Research, Cambridge, MA, USA). PCR was performed using a Smart Cycler II system (Cepheid, Sunnyvale, CA, USA) with 45 cycles of 95°C for 5 s and 55°C for 10 s; measurements were made at the end of a 60°C annealing step. Data were analyzed using Smart Cycler software (version 2.0d). All real-time PCR experiments were performed in quadruplicate. The calculated values for gene expression were normalized against the levels of glyceraldehyde-3phosphate dehydrogenase (GAPDH) mRNA at the same time point.

### 2.1. Chondrocyte Cell Culture

Chondrocytes derived from normal human femoral cartilage were obtained from Cell Applications (San Diego, CA, USA). The cells were maintained in Dulbecco's modified Eagle's medium/nutrient mixture F-12 (DMEM; Gibco BRL, Rockville, MD, USA) containing 10% (v/v) fetal bovine serum (FBS; HyClone Laboratories, Logan, UT, USA), 1% (v/v) insulin-transferrin-selenium-X supplement (Invitrogen, Carlsbad, CA, USA), and 1% (v/v) penicillin-streptomycin solution (Sigma Chemical, St. Louis, MO, USA) at 37°C in a humidified atmosphere under 5% CO_2_.

For treatment with IL-1*β* (Genzyme/Techne, Minneapolis, MN, USA), cells were seeded onto 100 mm tissue culture dishes at a density of 5 × 10^6^ cells/cm^2^. After overnight incubation, the cells were cultured for up to 28 days in DMEM containing 10% (v/v) or 2% (v/v) FBS with 0, 10, or 100 U/mL IL-1*β*, with or without 1 *μ*M celecoxib (Astellas Pharma, Tokyo, Japan). One unit (U) of IL-1*β* corresponds to the unit activity in the colorimetric MTT assay with CTLL-2 cells [26]. The IL-1*β* concentrations were identical to those used in our previous studies [19–21, 23], and similar to those found in inflammatory TMJ synovial fluid [15]. The celecoxib concentration used in this study was decided based on the report of Itthipanichpong et al. [25], who examined the blood level after injecting celecoxib, and our previous study, which found that IL-1*β* increases the production of PGE_2_, COX-2, and EP4 receptor in the same chondrocytes as used in this study [23]. Celecoxib specifically inhibits COX-2 [24, 25]. We have already confirmed that 1 *μ*M celecoxib blocked PGE_2_ production by inhibiting COX-2 in 100 U/mL IL-1*β*-stimulated chondrocytes [23].

### 2.2. Enzyme-Linked Immunosorbent Assay (ELISA)

Chondrocytes were incubated in DMEM containing 10% (v/v) FBS with 0, 10, or 100 U/mL IL-1*β* for 21 or 28 days. The culture medium was changed to DMEM containing 2% (v/v) FBS with 0, 10, or 100 U/mL IL-1*β*, and the cells were then cultured for 24 h at 37°C in a humidified atmosphere under 5% CO_2_.

The levels of M-CSF, RANKL, and OPG protein in the culture medium were determined using a commercially available ELISA kit (R&D Systems, Minneapolis, MN, USA) according to the manufacturer's instructions. Assays were performed in triplicate for each specimen and the data were converted to pg/mL or ng/mL.

### 2.3. Tartrate-Resistant Acid Phosphatase (TRAP) Staining of Osteoclast Precursors

Cells were cultured in DMEM containing 10% (v/v) FBS with or without 100 U/mL IL-1*β* for 7 or 28 days. The culture medium was changed to DMEM containing 2% (v/v) FBS without IL-1*β*, and the cells were incubated at 37°C in a humidified atmosphere under 5% CO_2_. The culture medium was collected after 24 h and diluted with an equal volume of DMEM containing 2% (v/v) FBS, supplemented with 50 ng/mL soluble RANKL (Wako Pure Chemical, Osaka, Japan), and then used as conditioned medium. The RANKL concentration used was equivalent to that used in a study examining the expression of enzymes related to bone resorption in RAW264.7 cells [27].

To examine the differentiation of osteoclasts, we used the murine monocyte/macrophage cell line, RAW264.7 (Dainippon Pharmaceutical, Osaka, Japan). RAW264.7 cells were plated into 96-well culture plates (6 × 10^3^ cells/cm^2^) and cultured in conditioned medium for 7 days. Cells cultured with two types of conditioned medium (from IL-1-untreated and -treated chondrocyte growth conditions) were fixed and stained using a TRAP and alkaline phosphatase double-staining kit (TaKaRa Bio) according to the manufacturer's protocol [28] on day 7 of culture. TRAP-positive multinucleate cells with more than three nuclei were considered to be osteoclast-like cells, and the data were converted into the percentage of TRAP-positive multinucleate cells of the total number of cells.

### 2.4. Statistical Analysis

All experiments were performed in triplicate or quadruplicate. Values shown represent the mean ± standard deviation (SD). Significant differences were detected using Student's *t*-test with Bonferroni's correction or analysis of variance (ANOVA), and *P*-values <  .05 were considered significant.

## 3. Results

### 3.1. RANKL Gene and Protein Expression in Chondrocytes

**Figure 1:**
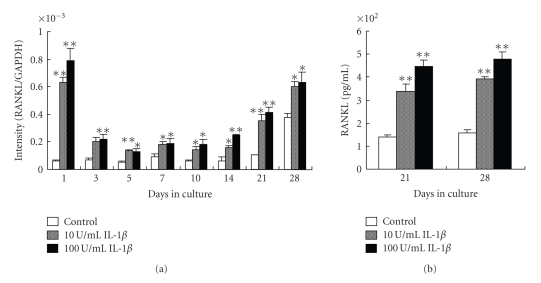
Effect of IL-1*β* on RANKL gene and protein expression. (a) Cells were cultured with 0, 10, or 100 U/mL IL-1*β* and RANKL gene expression was determined by real-time PCR on days 1, 3, 5, 7, 10, 14, 21, and 28 of culture. (b) RANKL protein expression determined by ELISA on days 21 and 28. Each bar indicates the mean ± SD from four separate experiments. *P < .05, **P < .01 for IL-1*β* treatment versus the control.

In the absence of IL-1*β*, gene expression was unchanged from day 1 to 21 of culture. In the presence of IL-1*β*, gene expression increased significantly compared to the control at each time point (Figure 1(a)). 

Consistent with the results obtained for gene expression, RANKL protein expression was significantly higher in the presence of IL-1*β* compared to untreated control cells on days 21 and 28 (Figure 1(b)).

### 3.2. M-CSF Gene and Protein Expression in Chondrocytes

**Figure 2:**
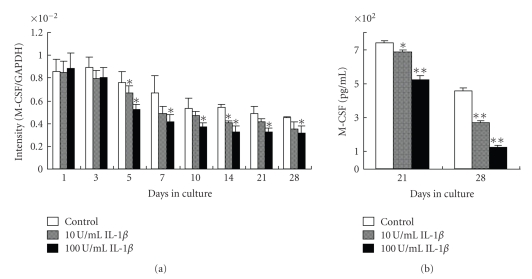
Effect of IL-1*β* on M-CSF gene and protein expression. (a) Cells were cultured with 0, 10, or 100 U/mL IL-1*β* and M-CSF gene expression was determined by real-time PCR on days 1, 3, 5, 7, 10, 14, 21, and 28 of culture. (b) M-CSF protein expression determined by ELISA on days 21 and 28. Each bar indicates the mean ± SD from four separate experiments. *P < .05, **P < .01 for IL-1*β* treatment versus the control.

Despite this general decreasing trend, M-CSF gene expression was consistently lower in IL-1*β*-treated cells compared to the untreated control after 5 days of culture (Figure 2(a)).

Consistent with the results obtained in the gene expression study, M-CSF protein expression was significantly lower in the presence of IL-1*β* compared to untreated control cells on days 21 and 28 (Figure 2(b)).

### 3.3. OPG Gene and Protein Expression in Chondrocytes

**Figure 3:**
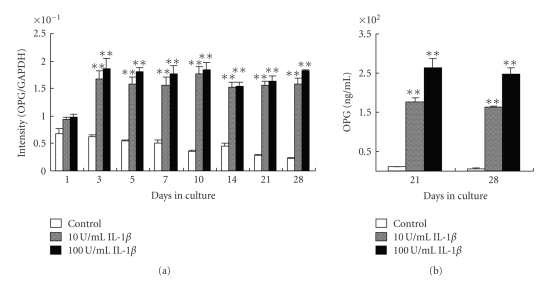
Effect of IL-1*β* on OPG gene and protein expression. (a) Cells were cultured with 0, 10, or 100 U/mL IL-1*β* and OPG gene expression was determined by real-time PCR on days 1, 3, 5, 7, 10, 14, 21, and 28 of culture. (b) OPG protein expression determined by ELISA on days 21 and 28. Each bar indicates the mean ± SD from four separate experiments. **P < .01 for IL-1*β* treatment versus the control.

In the absence of IL-1*β*, OPG gene expression decreased gradually over 28 days. In the presence of IL-1*β*, OPG mRNA expression was significantly higher than that in control cells at each time point (Figure 3(a)).

Consistent with the results obtained in the gene expression study, OPG protein expression was significantly higher in the presence of IL-1*β* compared to untreated control cells on days 21 and 28 (Figure 3(b)).

### 3.4. Effect of IL-1*β* and Celecoxib on RANKL, M-CSF, and OPG Gene Expression

**Figure 4:**
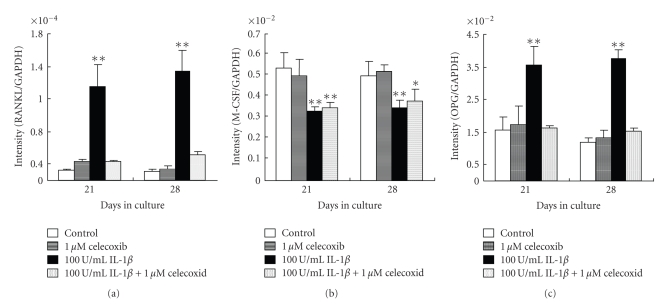
Effect of IL-1*β* and/or celecoxib on RANKL, M-CSF, and OPG gene expression. Cells were cultured with 0 or 100 U/mL IL-1*β*, with or without 1 *μ*M celecoxib, and RANKL, M-CSF, and OPG gene expression was determined by real-time PCR on days 21 and 28 of culture. Each bar indicates the mean ± SD from four separate experiments. *P < .05, **P < .01 for IL-1*β* treatment versus the control.

In the absence of IL-1*β*, RANKL, M-CSF, and OPG gene expression was unaffected by the presence of celecoxib. However, the presence of celecoxib suppressed the IL-*β*-induced increase in RANKL and OPG mRNA expression to control levels. In contrast, celecoxib did not affect the IL-1*β*-induced M-CSF gene expression on either of the days tested (Figure 4).

### 3.5. TRAP Staining of Osteoclast-Like Cells

**Figure 5:**
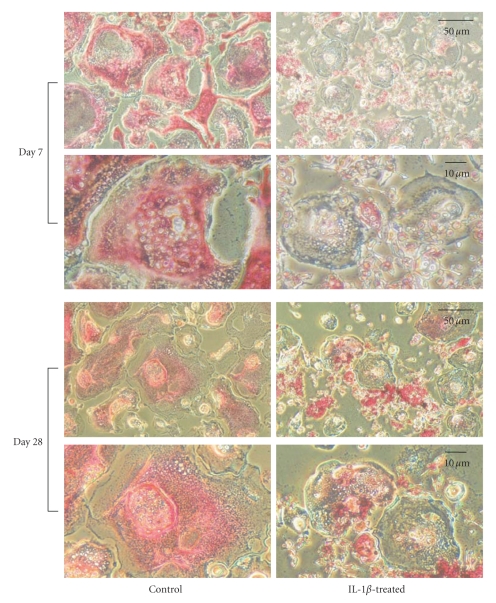
Effect of conditioned medium from IL-1*β*-treated and -untreated chondrocytes on the formation of osteoclast-like cells. Cells were cultured in DMEM containing 10% FBS with or without 100 U/mL IL-1*β* for 7 or 28 days. The culture medium was changed to DMEM containing 2% FBS without IL-1*β*, and the cells were cultured for 24 h. The culture medium was collected and diluted with an equal volume of DMEM containing 2% FBS, supplemented with 50 ng/mL soluble RANKL, and then used as conditioned medium. RAW264.7 cells, an osteoclast precursor, were cultured in conditioned media from IL-1*β*-treated and -untreated (control) chondrocytes for up to 7 days, and then stained using a TRAP and alkaline phosphatase double-staining kit.

**Figure 6:**
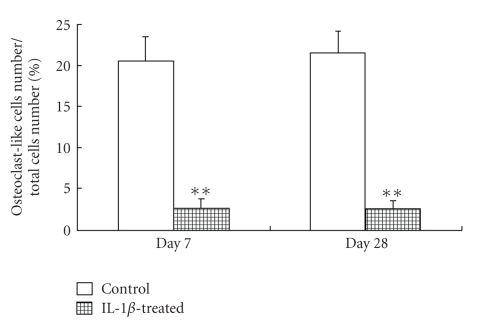
Effect of IL-1*β* on osteoclast differentiation estimated by the number of TRAP-positive multinucleate cells. Cells were cultured in DMEM containing 10% FBS with or without 100 U/mL IL-1*β* for 7 or 28 days. RAW264.7 cells were cultured under the conditions described in Figure 5. TRAP-positive multinucleate cells with more than three nuclei were considered osteoclasts, and the data were converted to the percentage of TRAP-positive multinucleate cells of the total number of cells. Each bar indicates the mean ± SD from separate experiment. **P < .01 for IL-1b treatment versus the control.

The indirect effect of IL-1*β* on the formation of osteoclast-like cells was examined using TRAP staining, a differentiation marker of osteoclasts. In this experiment, cells were cultured with soluble RANKL and conditioned medium from 100 U/mL IL-1*β*-treated or untreated chondrocytes for up to 7 or 28 days. Many TRAP-positive multinucleate cells were observed after culture with medium from IL-1*β*-untreated chondrocytes compared to cells cultured with medium from IL-1*β*-treated chondrocytes (Figure 5). The number of TRAP-positive multinucleate cells was significantly lower when the medium was conditioned by chondrocytes cultured with IL-1*β* (Figure 6). In addition, ratio of IL-1*β*-treated/control was almost the same in both on days 7 and 28 of culture: the ratio on days 7 and 28 of culture was 0.100 and 0.098, respectively.

## 4. Discussion

Cytokines released at sites of inflammation and infection can alter the normal processes of cartilage turnover, resulting in its pathological destruction or formation [29], and the levels of inflammatory cytokines are higher than normal in the synovial fluid of patients with TMJ arthrosis [30, 31]. IL-1*β* in synovial fluid not only plays a central role in the pathophysiology of cartilage damage and degradation, but is also associated with pain and hyperalgesia in TMJ.

Recently, we demonstrated that IL-1*β* increased the production of PGE_2_, COX-2, and PG receptor EP4 in human chondrocytes, suggesting that the IL-1*β* promotes the expression of EP4 by an autocrine mechanism involving increased PGE_2_ production in chondrocytes [23]. In light of this finding, we hypothesized that IL-1*β* promotes an imbalance in bone and cartilage matrix turnover through an autocrine mechanism involving PGE_2 _produced by chondrocytes. Therefore, we focused on the formation of osteoclasts via the RANK/RANKL/OPG system that communicates between osteoclast precursors and chondrocytes. This study was performed to clarify the effect of IL-1*β* and/or celecoxib, a specific inhibitor of COX-2 [24, 25], on the expression of M-CSF, RANKL, and OPG in human chondrocytes, and the indirect effect of IL-1*β* on the formation of osteoclast-like cells using RAW264.7 cells as osteoclast precursors. The chondrocytes used in this study strongly expressed type II collagen and aggrecan, which is the common phenotype expressed by proliferating chondrocytes and hypertrophic chondrocytes; whereas the expression of type X collagen, which is the phenotype expressed by hypertrophic chondrocytes, was very low in the cells (data not shown). Therefore, we consider that the chondrocytes used in this study were proliferating chondrocytes.

In designing these experiments, we first determined an appropriate length for the culture period. In many previous studies on M-CSF, RANKL, and OPG expression in chondrocytes, the culture periods were brief (24 h; [4, 5, 32–34]). However, because arthrosis of the TMJ typically exhibits chronic progression, we examined gene and protein expression in chondrocytes over a much longer culture period of 28 days. The IL-1*β* concentrations used in the current study are identical to those used in our previous studies [20, 21, 23]. The celecoxib concentration used was identical to that used in the previous studies [23, 25]. 

In this study, RANKL and OPG expression increased significantly in cells cultured with IL-1*β*, whereas M-CSF expression decreased. In addition, the level of OPG protein was markedly higher than that of RANKL in the cells. Given the important role of RANK, RANKL, and OPG in bone metabolism and immune function, it has been suggested that the RANK/RANKL/OPG system and cytokines may work together to cause bone resorption by regulating the RANKL/OPG ratio [35]. RANK, RANKL, and OPG mRNA were expressed in cartilage and bone [1, 4]. Deschner et al. [33] reported that fibrochondrocytes constitutively expressed low levels of RANKL, but high levels of OPG; the authors reported that IL-1*β* upregulated the expression and synthesis of RANKL; whereas OPG expression was unaffected after 4 and 24 hours. Komuro et al. [4] reported that OPG expression in chondrocytes increased in response to IL-1*β* stimulation for 24 h. In osteoblasts, RANKL expression increased after stimulation with IL-1, IL-6, and IL-11 [1]; whereas OPG expression decreased [1, 10]. On the other hand, M-CSF expression in osteoblasts increased after stimulation with IL-1*α* [10]; whereas the effect of IL-1*α* on M-CSF expression in chondrocytes was unclear. In this study, the expression patterns of RANKL, M-CSF, and OPG in the chondrocytes changed without IL-1*β* stimulation; in the long-term culture. In addition, we examined the expression of RANKL, M-CSF, and OPG after long term culture in the absence of IL-1*β* followed by short-term IL-1*β* stimulation. As a result, OPG and RANKL expression increased with IL-1*β* stimulation, whereas M-CSF expression decreased (data not shown). We believe that this change is related to cell differentiation. Previously, we showed that the alkaline phosphatase activity in the same chondrocytes increased gradually from days 7 to 28 of culture without IL-1*β* or IL-6 stimulation [20, 22], and that the activity decreased significantly from day 10 of culture with IL-1*β* stimulation compared with control [20]. In addition, the expression of type X collagen in the same chondrocytes increased gradually from day 14 of culture without IL-1*β*, and the expression decreased significantly on days 21 and 28 of culture with IL-1*β* stimulation compared with control (data not shown). These results suggest that proliferating chondrocytes differentiate gradually into hypertrophic chondrocytes in long-term culture. Therefore, our results suggest that IL-1*β* not only affected the expression of RANKL, M-CSF, and OPG in chondrocytes, but may also affect the course of cell differentiation. In addition, IL-1*β* showed the same effect for the expression of RANKL, M-CSF, and OPG for any culture period, while the control value changed in the long-term culture. Therefore, these previous studies suggest that the effect of IL-1 on RANKL expression is the same in osteoblasts and chondrocytes; whereas the effect of IL-1 on OPG and M-CSF expression differs according to cell type and IL-1 subtype. We will examine the reason why the effect of IL-1*β* on M-CSF expression differed between osteoblasts and chondrocytes in the future.

To clarify the indirect participation of PGE_2_ on the expression of RANKL, OPG, and M-CSF in chondrocytes, we examined the effect of IL-1*β* and/or celecoxib on cultured cells. According to our results, celecoxib, a specific inhibitor of COX-2 [24, 25], blocked the stimulatory effect of IL-1*β* on the expression of OPG and RANKL; whereas it did not affect the IL-1*β*-mediated reduction in M-CSF expression. Previously, we found that IL-1*β* stimulated the expression of the EP4 receptor via an autocrine mechanism involving PGE_2_ production in human chondrocytes [23]. The findings in this study also suggest that IL-1*β* induces the expression of OPG and RANKL in human chondrocytes via an autocrine mechanism that increases PGE_2_ production. Sakata et al. [5] reported that the IL-1*β*-induced increase in OPG levels in human periodontal ligament cells is suppressed through the de novo synthesis of PGE_2_. In contrast, Coon et al. [36] reported that the baseline levels of RANKL and OPG expression in COX-2 knockout osteoblasts decreased compared to wild-type osteoblasts. Considering our results, these findings suggest that the effect of PGE_2_ on OPG expression may differ according to cell type.

Tanabe et al. [2] reported that conditioned medium containing M-CSF and PGE_2_ produced by IL-1*α*-treated osteoblasts and soluble RANKL increased TRAP staining in RAW264.7 cells. Therefore, we conducted a similar experiment using human chondrocytes. According to our results, conditioned medium from IL-1*β*-treated chondrocytes decreased TRAP staining in RAW264.7 cells. These results suggest that the function of IL-1 in the differentiation of RAW264.7 cells via the RANK/RANKL/OPG system may differ between osteoblasts and chondrocytes.

In conclusion, our results suggest that IL-1*β* suppresses the formation of osteoclast-like cells via an increase in OPG production and a decrease in M-CSF production, at least in part, in the human chondrocytes used in this study, and that OPG production may increase through an autocrine mechanism involving celecoxib-related PGs. To reinforce this conclusion further, it is necessary to conduct a similar experiment using various chondrocytes, such as an established chondrocyte cell line, or other clinical specimens in the future.

## Figures and Tables

**Table 1:** PCR primers used in the experiments.

Target	Primer sequence (5′–3′)	GenBank Acc.
*RANKL*	TGCCAGTGGGAGATGTTAGAC	NM_03012.2
	CCTTCAATTGCGCTAGATGAC	
*M-CSF*	TAGCCACATGATTGGGAGTGGA	NM_172212.1
	CTCAAATGTAATTTGGCACGAGGTC	
*OPG*	AGCTGCAGTACGTCAAGCAGGA	NM_002546.3
	TTTGCAAACTGTATTTCGCTCTGG	
*GAPDH*	GCACCGTCAAGGCTGAGAAC	NM_001725661.1
	ATGGTGGTGAAGACGCCAGT	

## References

[1] Bezerra MC, Carvalho JF, Prokopowitsch AS, Pereira RMR (2005). RANK, RANKL and osteoprotegerin in arthritic bone loss. *Brazilian Journal of Medical and Biological Research*.

[2] Tanabe N, Maeno M, Suzuki N (2005). IL-1*α* stimulates the formation of osteoclast-like cells by increasing M-CSF and PGE_2_ production and decreasing OPG production by osteoblasts. *Life Sciences*.

[3] Lacey DL, Timms E, Tan H-L (1998). Osteoprotegerin ligand is a cytokine that regulates osteoclast differentiation and activation. *Cell*.

[4] Komuro H, Olee T, Kühn K (2001). The osteoprotegerin/receptor activator of nuclear factor *κ*B/receptor activator of nuclear factor *κ*B ligand system in cartilage. *Arthritis and Rheumatism*.

[5] Sakata M, Shiba H, Komatsuzawa H (2002). Osteoprotegerin levels increased by interleukin-1*β* in human periodontal ligament cells are suppressed through prostaglandin E_2_ synthesized de novo. *Cytokine*.

[6] Kishimoto K, Kitazawa R, Kurosaka M, Maeda S, Kitazawa S (2006). Expression profile of genes related to osteoclastogenesis in mouse growth plate and articular cartilage. *Histochemistry and Cell Biology*.

[7] Chin JE, Winterrowd GE, Krzesicki RF, Sanders ME (1990). Role of cytokines in inflammatory synovitis: the coordinate regulation of intercellular adhesion molecule 1 and HLA class I and class II antigens in rheumatoid synovial fibroblasts. *Arthritis and Rheumatism*.

[8] Strand V, Kavanaugh AF (2004). The role of interleukin-1 in bone resorption in rheumatoid arthritis. *Rheumatology*.

[9] Horiuchi T, Yoshida T, Koshihara Y (1999). The increase of parathyroid hormone-related peptide and cytokine levels in synovial fluid of elderly rheumatoid arthritis and osteoarthritis. *Endocrine Journal*.

[10] Tanaka Y, Nakayamada S, Okada Y (2005). Osteoblasts and osteoclasts in bone remodeling and inflammation. *Current Drug Targets: Inflammation & Allergy*.

[11] Foged J (1949). Temporomandibular arthrosis. *The Lancet*.

[12] Frenkel SR, Di Cesare PE (1999). Degradation and repair of articular cartilage. *Frontiers in Bioscience*.

[13] Poole AR (1999). An introduction to the pathophysiology of osteoarthritis. *Frontiers in Bioscience*.

[14] Mori H, Nishida K, Ozaki T, Inoue H, Nakanishi T (2008). Isolation of a mRNA preferentially expressed in synoviocytes from rheumatoid arthritis that is identical with lumican, which encodes a collagen binding extracellular matrix protein. *Journal of Hard Tissue Biology*.

[15] Alstergren P, Benavente C, Kopp S (2003). Interleukin-1*β*, interleukin-1 receptor antagonist, and interleukin-1 soluble receptor II in temporomandibular joint synovial fluid from patients with chronic polyarthritides. *Journal of Oral and Maxillofacial Surgery*.

[16] Takahashi T, Kondoh T, Fukuda M, Yamazaki Y, Toyosaki T, Suzuki R (1998). Proinflammatory cytokines detectable in synovial fluids from patients with temporomandibular disorders. *Oral Surgery, Oral Medicine, Oral Pathology, Oral Radiology, and Endodontics*.

[17] Alstergren P, Ernberg M, Kvarnström M, Kopp S (1998). Interleukin-1*β* in synovial fluid from the arthritic temporomandibular joint and its relation to pain, mobility, and anterior open bite. *Journal of Oral and Maxillofacial Surgery*.

[18] Kubota E, Imamura H, Kubota T, Shibata T, Murakami K-I (1997). Interleukin 1*β* and stromelysin (MMP3) activity of synovial fluid as possible markers of osteoarthritis in the temporomandibular joint. *Journal of Oral and Maxillofacial Surgery*.

[19] Aida Y, Maeno M, Ito-Kato E, Suzuki N, Shiratsuchi H, Matsumura H (2004). Effect of IL-1*α* on the expression of cartilage matrix proteins in human chondrosarcoma cell line OUMS-27. *Life Sciences*.

[20] Aida Y, Maeno M, Suzuki N, Shiratsuchi H, Motohashi M, Matsumura H (2005). The effect of IL-1*β* on the expression of matrix metalloproteinases and tissue inhibitors of matrix metalloproteinases in human chondrocytes. *Life Sciences*.

[21] Aida Y, Maeno M, Suzuki N (2006). The effect of IL-1*β* on the expression of inflammatory cytokines and their receptors in human chondrocytes. *Life Sciences*.

[22] Namba A, Aida Y, Suzuki N (2007). Effects of IL-6 and soluble IL-6 receptor on the expression of cartilage matrix proteins in human chondrocytes. *Connective Tissue Research*.

[23] Watanabe Y, Namba A, Honda K (2009). IL-1*β* stimulates the expression of prostaglandin receptor EP4 in human chondrocytes by increasing production of prostaglandin E_2_. *Connective Tissue Research*.

[24] Yoshino T, Kimoto A, Kobayashi S (2005). Pharmacological profile of celecoxib, a specific cyclooxygenase-2 inhibitor. *Arzneimittel-Forschung*.

[25] Itthipanichpong C, Chompootaweep S, Wittayalertpanya S (2005). Clinical pharmacokinetic of celecoxib in healthy Thai volunteers. *Journal of the Medical Association of Thailand*.

[26] Gillis S, Ferm MM, Ou W, Smith KA (1978). T cell growth factor: parameters of production and a quantitative microassay for activity. *Journal of Immunology*.

[27] Fujisaki K, Tanabe N, Suzuki N (2007). Receptor activator of NF-*κ*B ligand induces the expression of carbonic anhydrase II, cathepsin K, and matrix metalloproteinase-9 in osteoclast precursor RAW264.7 cells. *Life Sciences*.

[28] Burstone MS (1958). Histochemical demonstration of acid phosphatases with naphthol AS-phosphates. *Journal of the National Cancer Institute*.

[29] Shinoda C, Takaku S (2000). Lnterleukin-1 *β*, interleukin-6, and tissue inhibitor of metalloproteinase-l in the synovial fluid of the temporomandibular joint with respect to cartilage destruction. *Oral Diseases*.

[30] Sandler NA, Buckley MJ, Cillo JE, Braun TW (1998). Correlation of inflammatory cytokines with arthroscopic findings in patients with temporomandibular joint internal derangements. *Journal of Oral and Maxillofacial Surgery*.

[31] Alstergren P, Kopp S, Theodorsson E (1999). Synovial fluid sampling from the temporomandibular joint: sample quality criteria and levels of interleukin-1*β* and serotonin. *Acta Odontologica Scandinavica*.

[32] Campbell IK, Ianches G, Hamilton JA (1993). Production of macrophage colony-stimulating factor (M-CSF) by human articular cartilage and chondrocytes. Modulation by interleukin-1 and tumor necrosis factor *α*. *Biochimica et Biophysica Acta*.

[33] Deschner J, Wypasek E, Ferretti M, Rath B, Anghelina MA, Agarwal S (2006). Regulation of RANKL by biomechanical loading in fibrochondrocytes of meniscus. *Journal of Biomechanics*.

[34] Quintero M, Riera H, Colantuoni G (2008). Granulocyte-macrophage colony stimulating factor is anabolic and interleukin-1*β* is catabolic for rat articular chondrocytes. *Cytokine*.

[35] Saidenberg-Kermanac’h N, Cohen-Solal M, Bessis N, De Vernejoul M-C, Boissier M-C (2004). Role for osteoprotegerin in rheumatoid inflammation. *Joint Bone Spine*.

[36] Coon D, Gulati A, Cowan C, He J (2007). The role of cyclooxygenase-2 (COX-2) in inflammatory bone resorption. *Journal of Endodontics*.

